# Diffractive optical computing in free space

**DOI:** 10.1038/s41467-024-45982-w

**Published:** 2024-02-20

**Authors:** Jingtian Hu, Deniz Mengu, Dimitrios C. Tzarouchis, Brian Edwards, Nader Engheta, Aydogan Ozcan

**Affiliations:** 1 Electrical and Computer Engineering Department, University of California, Los Angeles, CA 90095 USA; 2 Bioengineering Department, University of California, Los Angeles, CA 90095 USA; 3 California NanoSystems Institute (CNSI), University of California, Los Angeles, CA 90095 USA; 4 Electrical and Systems Engineering, University of Pennsylvania, Philadelphia, PA 19104 USA; 5 Meta Materials Inc., Athens, 15123 Greece

**Keywords:** Optics and photonics, Mathematics and computing

## Abstract

Structured optical materials create new computing paradigms using photons, with transformative impact on various fields, including machine learning, computer vision, imaging, telecommunications, and sensing. This Perspective sheds light on the potential of free-space optical systems based on engineered surfaces for advancing optical computing. Manipulating light in unprecedented ways, emerging structured surfaces enable all-optical implementation of various mathematical functions and machine learning tasks. Diffractive networks, in particular, bring deep-learning principles into the design and operation of free-space optical systems to create new functionalities. Metasurfaces consisting of deeply subwavelength units are achieving exotic optical responses that provide independent control over different properties of light and can bring major advances in computational throughput and data-transfer bandwidth of free-space optical processors. Unlike integrated photonics-based optoelectronic systems that demand preprocessed inputs, free-space optical processors have direct access to all the optical degrees of freedom that carry information about an input scene/object without needing digital recovery or preprocessing of information. To realize the full potential of free-space optical computing architectures, diffractive surfaces and metasurfaces need to advance symbiotically and co-evolve in their designs, 3D fabrication/integration, cascadability, and computing accuracy to serve the needs of next-generation machine vision, computational imaging, mathematical computing, and telecommunication technologies.

## Abstract (web summary)

Optical computing via free-space-based structured optical materials allows to access optical information without the need for preprocessing or optoelectronic conversion. In this Perspective, the authors describe opportunities and challenges in their use for optical computing, information processing, computational imaging and sensing.

## Introduction

**Fig. 1:**
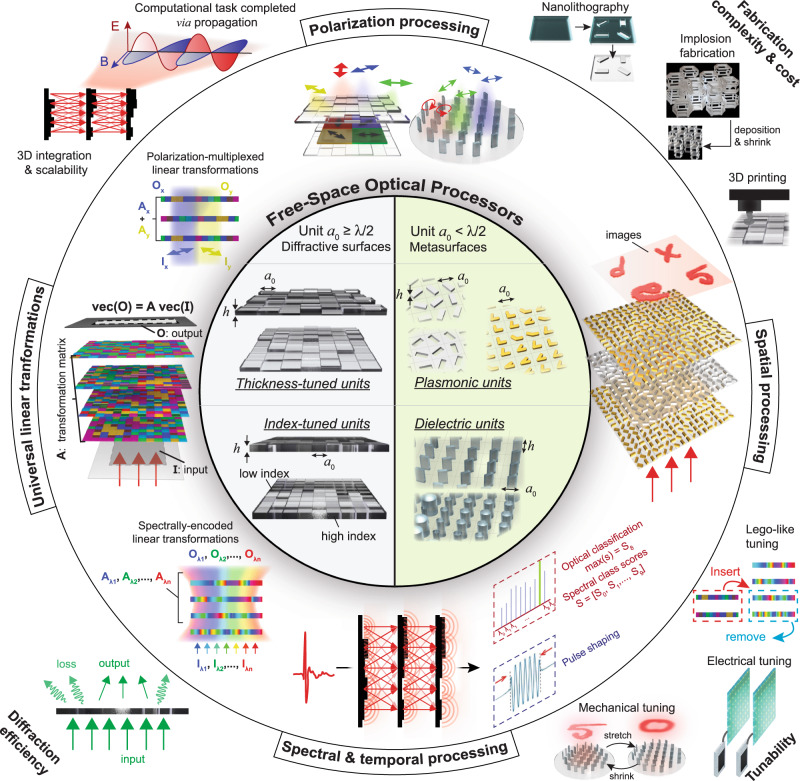
Overview of diffractive and metasurface systems for optical computing. Diffractive surfaces consist of thickness- and/or index-tuned units (λ/2 or larger) while metasurfaces consist of substructured metallic and/or dielectric units below λ/2. These free-space processors can perform polarization processing, spatial processing, universal linear transformations, and spectral & temporal processing of waves.

The past decade has witnessed an increasing interest in optical computing platforms because of their potential to realize fast, massively parallel computation at low power consumption^1–4^. For example, integrated photonics-based processors^5–9^ with on-chip interferometers and waveguide-embedded light sources were designed to replace or empower their electronic counterparts. These planar optoelectronic architectures can implement various linear and non-linear operations and can rapidly self-tune and reconfigure, making them powerful and versatile for optoelectronic computing^10,11^. Therefore, integrated photonics-based processors have advantages in their reconfigurability and ease of integration with electronics, making them ideal for task-specific on-chip accelerators. However, integrated photonics devices often require preprocessing of input information, for example, to retrieve the lost phase information of an input scene due to intensity-only detection at the optoelectronic sensor array or simply to vectorize the multi-dimensional image of an input object into planar signals. In contrast, free-space optical processors (Fig. 1) can perform a given computation or inference task via light propagation and diffraction through a series of structured surfaces. Without any optoelectronic conversion or preprocessing of input information, diffractive optical processors perform computational tasks with direct access to all the encoded optical information in 3D, including the spatial phase and amplitude, polarization, spectrum, and orbital angular momentum (OAM) information of the input wave, which represents an object or a scene. This direct access to all the degrees of freedom carried by the input waves provides significant advantages to free-space optical processors for various applications such as all-optical statistical inference, wireless telecommunications, and computational imaging and sensing^12^, where the information is created/represented and transmitted through electromagnetic waves. Therefore, free-space optical processors offer advantages in visual information processing and related applications due to their direct access to 2D or 3D optical information. Driven by some of these fundamental advantages, free-space optical computing systems (Fig. 1) have undergone a paradigm shift in their designs facilitated by emerging deep-learning methods and unique fabrication technologies.

This Perspective focuses on data-driven design and fabrication of (1) diffractive surfaces that are structured at the wavelength scale (with a unit size of *λ*/2 or larger), and (2) metasurfaces with deeply subwavelength dielectric or metallic units (smaller than *λ*/2). Considering only the propagating electromagnetic waves and ignoring the evanescent fields, the lateral footprint of photons in free-space is ~*λ*/2; the optical processor design spaces covered by diffractive surfaces and metasurfaces intersect at this *λ*/2 boundary, giving us a plethora of transformative opportunities to control and process light at different scales. For example, diffractive optical processors constructed based on a series of densely-packed surfaces engineered through deep-learning can achieve universal linear transformations within a compact diffractive volume by approximating any arbitrary complex-valued matrix operation with an arbitrarily small error bound^13,14^; such diffractive processors also enable all-optical image classification using light diffraction through structured surfaces. In addition to directly processing the spatial phase and amplitude information of an input scene, diffractive optical processors can also be trained using deep-learning to exploit other information channels, including polarization^15^ and spectrum^16–18^, massively increasing the parallelism and bandwidth of free-space optical computing. Replacing or augmenting dielectric-based diffractive surfaces with metasurfaces can provide some additional degrees of freedom, including the engineering of dispersion, polarization, spin, and OAM, to further enhance the inference capacity and the computational power of free-space optical processors.

**Fig. 2:**
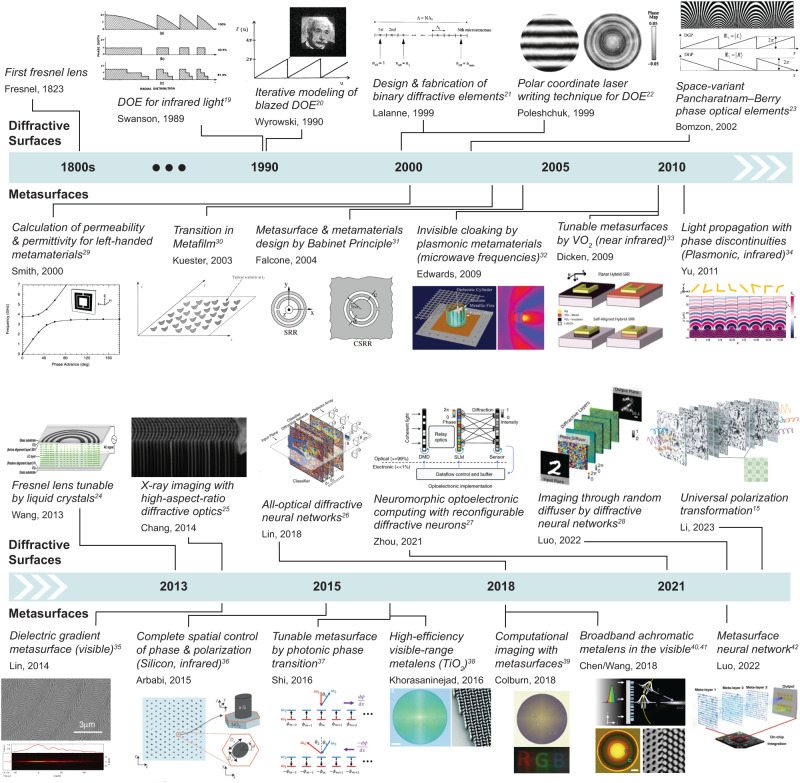
Timeline of diffractive^15,19–28^ and metasurface^29–42^ based optical devices and systems. Images are adapted from refs. ^26,39,91^ with permissions from AAAS, refs. ^20–24,33,95,162^ with permissions from © The Optical Society, refs. ^25,27,36,40,41^ with permissions from Springer Nature, refs. ^28,42^ under CC BY 4.0, ref. ^29^ with permissions from AIP publishing, and refs. ^31,32,37^ with permissions from APS.

Both diffractive surfaces and metasurfaces have received major research interest in the past decades (Fig. 2)^15,19–42^. Despite this tremendous progress, the synergy between the two frameworks in the context of free-space optical computing has not been explored in depth. This Perspective focuses on this emerging opportunity for diffractive surfaces and metasurfaces to symbiotically shape the future designs of free-space optical computing devices and technologies that will impact various applications such as optical machine learning, statistical inference, computational camera and microscope design, and telecommunications, among many others. As part of this Perspective’s comparative analysis of free-space diffractive computing (Fig. 1), we will also discuss four grand challenges ahead of this field of research:

1. Limits of computational accuracy and statistical inference capability of diffractive optical processors.
2. Dynamic reconfigurability for on-demand tuning of the optical processor function.
3. Speed, ease, and scalability of diffractive processor device fabrication, alignment, and integration, important for cost-effective and large-scale manufacturing and adoption of free-space optical processors.
4. Diffraction efficiency and energy consumption of the optical processor.

This Perspective focuses on these emerging opportunities and challenges centered around optical computing in free-space. We expect the comparatively analyzed methodologies in this Perspective to profoundly impact the next-generation wave-based free-space computing technologies ranging from all-optical statistical inference and computational imaging to wireless communications, edge computing, and others.

## Design principles of spatially-structured surfaces for computing

### Diffractive surfaces

**Fig. 3:**
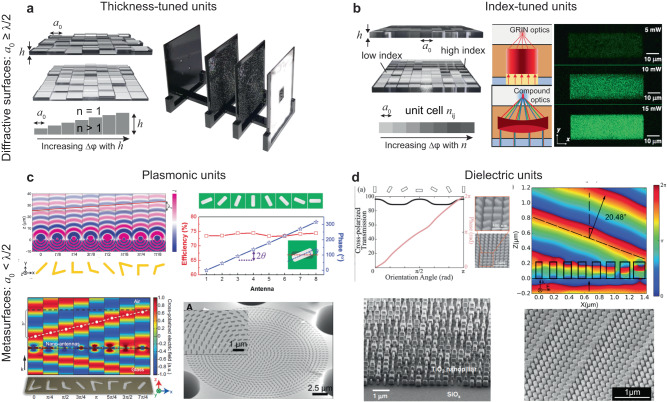
Design of diffractive surfaces and metasurfaces. Diffractive unit cell designs and their principles of wavefront modulation based on (**a**) thickness-tuning^26^ and (**b**) index-tuning^47,48^. Metasurface unit cell designs using (**c**) plasmonic^34,57,112^ and (**d**) dielectric materials^42,61,62^. **b** This is adapted with permission from ref. ^48^ by CC BY 4.0. **c** This is adapted with permission from ref. ^34^ by AAAS, refs. ^34,57^ by CC BY 4.0 and ref. ^112^ by ACS. **d** This is adapted with permission from ref. ^42^ by CC BY 4.0, ref. ^62^ by John Wiley and Sons and ref. ^61^ by ACS.

A diffractive surface-based free-space optical processor is spatially engineered at the wavelength scale (*λ*/2 or larger) to generate a desired spatial distribution of the complex-valued light transmission/reflection coefficients (Fig. 3a, b, Supplementary Fig. S1a). Unlike standard refractive optical components, e.g., lenses, such diffractive surfaces are pixelated in 2D, and their structure contains physical thickness and/or transmission discontinuities with a lateral period of ≥*λ*/2; also see Supplementary Discussion 1. The structure of these variations along the optical axis is typically on the order of the wavelength of the propagating light, and therefore, they can, in general, be modeled as 2D thin optical elements. Accordingly, the relationship between the incident monochromatic complex light field, ${U}_{{in}}(x,y)$, and the outgoing wavefront, ${U}_{{out}}(x,y)$, transmitted by a diffractive surface can be written as ${U}_{{out}}\left(x,y\right)=T(x,y){U}_{{in}}(x,y)$ with $T(x,y)$ denoting the 2D complex-valued transmission function of the diffractive surface at *λ*. The mathematical form of $T(x,y)$ depends on the physical mechanism of the optical light modulation and the related fabrication techniques.

In one simple form, light modulation can be achieved via amplitude modulation by partially reflecting and/or absorbing the light incident on certain pixels while allowing transmission on others. In this case, the modulation function $T(x,y)$ is real-valued, for example, binary (0 vs. 1 transmission). While some early research on diffractive optics heavily relied on such amplitude-only surface designs, they present limited diffraction efficiencies. Another commonly used diffractive surface design approach relies on the difference between the refractive indices of the fabrication material (substrate), ${n}_{{surface}}$ and the light propagation medium, ${n}_{{medium}}$, e.g., ${n}_{{medium}}=1$ in air. In general, the index contrast of a diffractive feature (with a height of *h*) with respect to the background induces an optical path delay in the form of $\Delta \varphi=\frac{2\pi h}{\lambda }({n}_{{surface}}-{n}_{{medium}})$. Typically, diffractive surfaces fabricated using a single dielectric material have a constant ${n}_{{surface}}$ over their pixels, which requires the thickness of the fabrication material to be a function of space, i.e., $h(x,y)$, leading to a 2D transmission function, $T\left(x,y\right)=\exp (j\frac{2\pi h(x,y)}{\lambda }({n}_{{surface}}-{n}_{{medium}}))$ (Fig. 3a). With the wide availability of additive manufacturing and nanofabrication techniques^43–46^, this approach has become one of the most commonly employed methods in implementing diffractive surfaces. An alternative method relies on the spatial engineering of the refractive index, i.e., ${n}_{{surface}}(x,y)$, while keeping $h$ constant (shown in Fig. 3b)^47,48^. This approach leads to a 2D transmission function in the form of $T\left(x,y\right)=\exp (j\frac{2\pi h}{\lambda }({n}_{{surface}}(x,y)-{n}_{{medium}}))$.

When the optical attenuation induced by the diffractive material is not negligible, i.e., ${n}_{{surface}}(x,y)=\kappa (x,y)+j\tau (x,y)$ is complex-valued, then $T\left(x,y\right)$ applies both an amplitude and a phase modulation at every unit cell of its design. For instance, assuming $\kappa \left(x,y\right)=\kappa$, $\tau \left(x,y\right)=\tau$ and ${n}_{{medium}}=1$, $T\left(x,y\right)$ can be written as $T\left(x,y\right)=\exp (j\frac{2\pi h(x,y)}{\lambda }({n}_{{surface}}-1))=\exp (-\frac{2\pi h(x,y)}{\lambda }(\tau ))\exp (j\frac{2\pi h(x,y)}{\lambda }(\kappa -1))$. This strategy for the modulation of the phase and amplitude of each diffractive unit cell, however, does *not* tailor the two variables independently because they are both controlled by $h(x,y)$. Independent control over both the amplitude and phase channels of each transmission function might be realized, for example, by spatial engineering of the material thickness and the complex-valued refractive index at the wavelength scale, covering thousands of individual unit cells over a diffractive surface. In fact, this is an area where metasurfaces can offer some additional degrees of freedom, which will be discussed next.

### Metasurfaces

Optical metasurfaces^23,30,38,49–52^ are planar structured surfaces consisting of unit cells with subwavelength structures that tailor the optical wavefront by their material properties, size, and shape^53,54^. Based on the target wavelength range of interest, the meta-units can be made of plasmonic metals (i.e., Au, Ag, and Al) or dielectric materials (i.e., Si, GaN, and TiO_2_). Typically, Al and Ag are the choices of plasmonic materials for ultraviolet and visible wavelengths where Au exhibits large non-radiative losses due to interband transitions^55,56^. Au exhibits lower losses at longer wavelengths ranging from the near-infrared to the terahertz range^55^. In the visible and near-infrared parts of the spectrum, plasmonic meta-units are typically formed by metallic nanoparticles (NPs) with various sizes and shapes and their inverted nanohole structures based on Babinet’s principle (Fig. 3c, Supplementary Fig. S1b)^31^. V-shaped meta-units tailor the wavefront by simultaneously exciting a symmetric resonant mode along the diagonal axis and an antisymmetric mode. The scattered light from each meta-unit exhibits a field amplitude and phase determined by the length $l$ of each rod and the angle Δ between them (Fig. 3c, left)^34^. In contrast, a rectangular plasmonic NP^34^ or nanohole^57^ oriented at an arbitrary angle $\theta$ can manipulate the scattered phase by the Pancharatnam–Berry (P-B) principle^58^, where the incident light with left- or right-hand circular polarization is converted to its orthogonal circular polarization state with an additional phase delay of 2*θ* on the transmitted beam (Fig. 3c, right). For operation in the gigahertz to terahertz range, other meta-units with more sophisticated geometries, such as C- and H-shaped elements, were also used to achieve higher cross-polarization conversion efficiencies^59^. Compared to dielectric materials, metallic meta-units, in general, allow an easier fabrication process based on lithography and lift-off processes^53^. One of the main limitations of plasmonic materials for building metasurface-based free-space optical processors is that their large losses can degrade the output diffraction efficiency drastically, also limiting the cascadability of such surfaces to enhance their function approximation power.

Partially motivated to solve this limitation, dielectric metasurfaces were developed to achieve better diffraction efficiencies in the near-infrared and visible wavelengths^38,60^. Typically, high aspect ratio meta-units of TiO_2_^38,42^ and GaN^40^ are used in the visible range and Si meta-units^61^ are used for near-infrared applications. Similar to their plasmonic counterparts, dielectric meta-units with rectangular or elliptical shapes can tailor the wavefront phase of circularly-polarized input light by tuning their in-plane orientations (Fig. 3d, left)^36,62^. This polarization selectivity of dielectric metasurfaces is also an effective tool for manipulating the spin states of light and has enabled spin to OAM conversion with high efficiency^63^. Polarization-insensitive phase responses can be achieved by using high-aspect-ratio dielectric posts by adjusting their diameters (Fig. 3d, right). To cover the 0-2π phase range by diameter tuning, the dielectric phase elements need to be tall, typically larger than 400 nm^64^. The processing of dielectric metasurfaces generally relies on conformal deposition techniques such as atomic layer deposition (ALD)^65^ and chemical vapor deposition (CVD)^36^, which are slow, expensive, and incompatible with lift-off-based fabrication processes. Instead, anisotropic dry etching steps are often required to produce the high aspect ratio nanoscale meta-units. These fabrication challenges and others will be discussed in Section “Fabrication complexity and 3D alignment requirements”.

To provide a comprehensive overview of existing free-space optical devices, we summarized in Table 1 the material compositions and fabrication methods of diffractive surfaces and metasurfaces along with their optical properties, including efficiency, spectral bandwidth, reconfigurability, and polarization responsivity. These performance metrics are also critical for designing free-space optical computing systems and are often the determining factors for practical applications. Strategies to tackle the existing challenges and future opportunities to further improve diffractive optical processors and metasurfaces will be discussed in Section “Grand Challenges in Free-space Optical Computing and Performance Limitations”.

**Table 1:** Survey of various diffractive surface- and metasurface-enabled designs

	Operation Wavelength/Task		Diffractive Surfaces		Metasurfaces	
			Thickness-tuned units	Index-tuned units	Plasmonic/Metallic	Dielectric
Fabrication Methods	GHz-THz		3D printing (Stereolithography, Polyjet, etc.)	3D printing^190^	Photolithography^191^ or Laser writing^192^	Photolithography^193^
	Visible-NIR		Nanoscribe^136^, etc.	Implosion Fabrication^137^	EBL^38^ or DUV^194^	EBL/DUV/Nanoscribe^195^
Materials	GHz-THz		Resin (Veroblack^26^, etc.)	Polystyrene^196^	Au, IST^192^, etc.	Si^197^, etc.
	Visible-NIR		Resin (IP-162^136^, etc.)	Hydogel^137^ or SiO_2_-TiO_2_^198^	Au^103^, Al^112^, Ag, etc.	TiO_2_^38^, GaN^40^, Si^36^, etc.
Power Efficiency	GHz-THz	Single wavelength	26% (classification)^66^	31-72% (lens)^199^	50% (beam steering)^200^	68% (lens)^197^
		Broadband	76–91% (unidirectional imager)^201^	N/A	N/A	30–68% (lens)^202^
	Visible-NIR	Single wavelength	99%^203^	75% (lens)^204^	80% (reflection, holography)^98^	86% (lens)^38^
		Broadband	95% (lens)^205^	N/A	8.4–11%^206^	40%^40^
Reconfigurability	GHz-THz		Layer swapping^18^	N/A	Electrically-tuned^121,207^ (single-unit-level 0–2π phase programmability)	Optical pumping tuned by DMD^208^
	Visible-NIR		Spatial light modulator^27^	N/A	Substrate stretching^103^	1D liquid crystal supercells^100^
Spectral Bandwidth	GHz-THz	Lens	0.3–1.5 THz^209^	0.4–0.6 THz^210^	4.2–4.5 THz^211^	0.3–0.8 THz^202^
		Imaging, Beam steering, Linear transformations	0.37–0.44 THz (unidirectional imaging)^201^	N/A	0.4–1 THz (beam steering)^212^	0.6–1 THz (beam steering)^193^
			0.34–0.41 THz (multi-channel linear transformation)^17^			
	Visible-NIR	Lens	425–700 nm^205^	488–1680 nm^213^	1200–1680 nm^206^	400–660 nm^40^
		Holography	532–1550 nm^214^	N/A	630–1050 nm^98^	1.1–1.4 μm^215^
		Beam steering	450–950 nm^216^	N/A	480–660 nm^217^	1.4–1.8 μm^218^
Polarization Responses	GHz-THz		Multi-pixel universal polarization transformations^15^	N/A	Single-pixel polarization conversion^219^	N/A
	Visible-NIR		N/A	N/A	Versatile single-pixel polarization generation^112^	Subwavelength single-pixel polarization conversion^36^
						Color holography^220^

## Computing capabilities of free-space optical processors using structured surfaces

### Statistical inference and data classification

Deep-learning enabled optical networks of diffractive-^26,66,67^ and meta-units^42,62^ over a series of passive surfaces have emerged as all-optical machine learning platforms demonstrating promising capabilities in various visual inference tasks, e.g., all-optical object detection and classification referred to as diffractive deep neural networks (D^2^NN). Similar to electronic neural networks, diffractive optical networks also serve as parameterized function approximators. However, they process analog optical waves of a scene propagating through structured/engineered surfaces to perform statistical inference, instead of digitized signals inside a computer. While the trainable parameters of this optical diffractive black-box are the transmittance (and/or reflectance) coefficients, ${\alpha }_{i}\exp \left(j{\beta }_{i}\right)$ of the diffractive unit cells in a predetermined coordinate, $\left({x}_{i},{y}_{i},{z}_{i}\right),$ within the computing volume, the complex-valued weights of the connections between these diffractive features are not individually trainable, directly dictated by the light diffraction and the axial spacing between the diffractive layers. Currently, the optimization of diffractive optical networks for a given inference task uses deep-learning-based training implemented in a digital computer. The transmittance coefficients of the diffractive units within the free-space optical processor are updated using the associated gradients with respect to a penalty term (training loss function) that is specifically devised for the targeted machine learning task and the associated detector configuration at the output plane. Following the training phase, the resulting diffractive surfaces are fabricated and assembled to form the physical diffractive optical processor, which performs the target statistical inference task without any external computing power, except for the illumination light.

The early experimental demonstrations of diffractive optical networks were performed based on 3D-printed diffractive surfaces operating at THz wavelengths with e.g., 0.2 million phase-only diffractive features distributed over five dielectric diffractive surfaces^26^. Since these initial proof-of-concept demonstrations, various design advances have been reported leading to >98% and >90% all-optical blind classification accuracies on benchmark datasets of handwritten digits (MNIST) and fashion products (Fashion-MNIST), respectively^66,68^. The inference and generalization capabilities of diffractive classifiers can further be improved by exploiting machine learning methods that evoke collaboration among multiple classification networks. Optical classification systems relying on multiple class-specific diffractive optical networks exemplify such a design strategy^68^. As an alternative to these jointly-optimized class-specific diffractive network systems, one can also use ensemble learning techniques^69,70^ to advance the optical inference performance of diffractive processors; these approaches will be further discussed in Section “Grand Challenges in Free-space Optical Computing and Performance Limitations”.

Beyond these all-optical image classifiers comprised of dielectric diffractive surfaces, it is also possible to extend the D^2^NN framework to design metasurface-enabled diffractive optical image classification networks^42^. An on-chip metasurface-based diffractive object classifier operating at visible wavelengths was also demonstrated^42^. With meta-atoms containing TiO_2_ nanopillars placed on top of a SiO_2_ substrate, the physical (trainable) parameters of this diffractive network were set as the width of the TiO_2_ nanopillars in *x* and *y* directions, which controls the effective refractive index seen by the *x*- and *y*-polarized light waves. The experimentally demonstrated system was restricted to a single metasurface with 280×280 meta-atoms, optically classifying 4 data classes from each of MNIST and Fashion-MNIST datasets, encoded into two orthogonal polarization states^42^; with deeper architectures involving more metasurfaces, one following another, further advances in the inference accuracy spanning a larger number of data classes can be achieved.

The function approximation capabilities of free-space optical processors are not limited to statistical inference or classification tasks and can be extended to all-optically performing other general-purpose computational tasks, including e.g., logic operations and diffractive NAND gates that can be optically-cascaded^71,72^.

In the examples discussed so far, the diffractive optical networks, whether comprised of dielectric diffractive layers or metasurfaces, employed linear materials and lacked any form of nonlinearity except for the detector plane. In general, the statistical inference accuracy and function approximation capability of diffractive processors would benefit from power efficient and scalable integration of nonlinear optical processes as part of the free-space diffractive processor volume (see e.g., ref. ^73^), and this grand challenge will be discussed under Section “Grand Challenges in Free-space Optical Computing and Performance Limitations”.

### Universal linear transformations

Optical networks have been broadly used to perform matrix operations, e.g., $y={Ax}$, where A is an arbitrary complex-valued matrix to be approximated by the optical network. Such universal linear transformations have been realized by integrated photonic circuits consisting of waveguides connected with e.g., Mach-Zehnder interferometers^5,8^ as well as by free-space diffractive networks^13,14^. An integrated photonic waveguide-based crossbar array also realized universal matrix multiplications, achieving trillions of multiply-accumulate operations per second^74^. One difference between these two main-stream approaches (integrated photonic circuits vs. free-space) is that the latter can directly act on the 2D or 3D optical information of an object, without the need to vectorize or pre-process the input optical information, and therefore provides a better fit for direct analog processing of visual information contained in a scene.

**Fig. 4:**
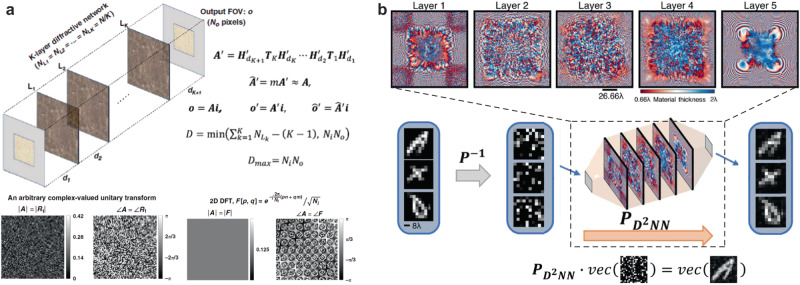
Diffractive optical networks can perform universal linear transformations. Diffractive surfaces trained to perform (**a**) linear operations including an arbitrary complex-valued transform, discrete Fourier transform (DFT)^13^ and (**b**) permutation operation^82^. Extensions of this framework to perform universal linear transformations under spatially and/or temporally incoherent illumination were also demonstrated^17,83^. **a**, **b** These are adapted with permission from ref. ^13^ and ref. ^82^, respectively, by CC BY 4.0.

The all-optical transformation by a diffractive free-space processor can be modeled as a complex-valued linear matrix multiplication operation between the input and output wavefront fields. It has been shown that diffractive optical processors are universal analog computing platforms for approximating arbitrary linear transformations^13,14,75,76^ which form the fundamental building blocks in a plethora of computational applications from communications^77,78^ and image processing^79,80^ to machine learning^81^ and beyond. The universality of deep-learning-based diffractive processor designs in all-optical implementations of complex-valued linear transformations has been demonstrated through numerical studies conducted on various types of linear operators including, (1) arbitrarily chosen complex-valued unitary, nonunitary, and noninvertible linear matrices (Fig. 4a)^13^, (2) 2D discrete Fourier transformation, (3) arbitrary 2D permutation operations (Fig. 4b)^82^, and (4) high-pass filtered coherent imaging^13^. While these results involved spatially and temporally coherent illumination, free-space-based diffractive processors were also designed to implement arbitrary linear transformations in optical intensity using spatially incoherent light^83^; furthermore, wavelength-multiplexed and massively parallel universal linear transformations were also demonstrated using deep-learning-designed diffractive processors, simultaneously covering hundreds of wavelength channels^17^.

In general, deeper diffractive architectures where a given number of diffractive degrees of freedom (*N*) is distributed across one diffractive surface following another present better optical approximation accuracies with improved output diffraction efficiencies compared to shallower architectures where the same *N* degrees of freedom are distributed to a smaller number of diffractive layers. Before the demonstration of this depth feature for universal linear transformations^75^, its first empirical evidence was presented for image classification tasks^26^, where deeper diffractive processors generalized to classify unknown object images better than shallower diffractive architectures.

An experimental demonstration of all-optical linear transformations e.g., an arbitrary permutation matrix with 625 input-output connections, was reported based on diffractive free-space processors comprised of *K* = 3 dielectric modulation surfaces operating at THz wavelengths^82^. Deep learning-designed diffractive networks were also used to experimentally realize data class-specific all-optical transformations and image encryption^84^. In these diffractive designs, the visual information that is encoded into the intensity of the optical field was all-optically encrypted by a data class-specific diffractive network that implemented a separate transformation matrix for each data class—i.e., a different linear transformation for each distinct class of objects, performed by the same diffractive optical processor^84^. This all-optical data-class-specific transformation system was experimentally demonstrated in different parts of the electromagnetic spectrum, i.e., 1550 nm and 0.75 mm wavelengths, and can enable fast, energy-efficient data encryption using a broad range of optical sources.

As another demonstration in the visible spectrum, a single-layer Si metasurface that could represent a linear complex-valued S-matrix was reported for solving the Fredholm integral equations^85^. This metagrating structure can approximate a Neumann series based on successive reflection between the metasurface and a semitransparent mirror. This analog computing platform that operates in the visible spectrum enables a highly compact and thin device that can achieve high processing speeds, with the possibility of on-chip integration.

### Optical processing of spatial information and computational imaging

Diffractive networks that process spatially encoded information of light present some unique opportunities for computational imaging and display systems. For example, imaging through unknown, random scattering, and diffusive media is a challenging problem of major significance for various fields ranging from biomedical imaging^86^ to autonomous robotic systems^87–89^. Diffractive networks provide a reliable all-optical solution to this challenge of imaging through unknown random diffusers by learning from the scattering process of images propagating through a large number of diffusers in a deep-learning-based training process^28^. Remarkably, this technique shows strong generalizability to unknown diffusers with a comparable correlation length to the diffusers used during the training. Diffractive surfaces are also capable of performing selective imaging, where only the desired classes of objects are recorded while other information is all-optically erased by the diffractive system^90^.

Diffractive networks also provide a powerful tool to achieve pixel super-resolution that can contribute to the development of high-resolution displays using spatial light modulators (SLMs) with limited pixel density and space-bandwidth products (SBP)^91^. The resolution of the displayed images can be enhanced up to 4-fold by (1) encoding the high-resolution images into low-resolution SLM patterns using a convolutional neural network (CNN) and (2) decoding the low-resolution images into the high-resolution images by a jointly-trained diffractive network^91^. This all-optical pixel super-resolution system can surpass the SBP restrictions of the wavefront modulator while maintaining its image field-of-view. The method can also significantly reduce the data transmission and storage burden by encoding the high-resolution input images into compact, low-resolution representations.

**Fig. 5:**
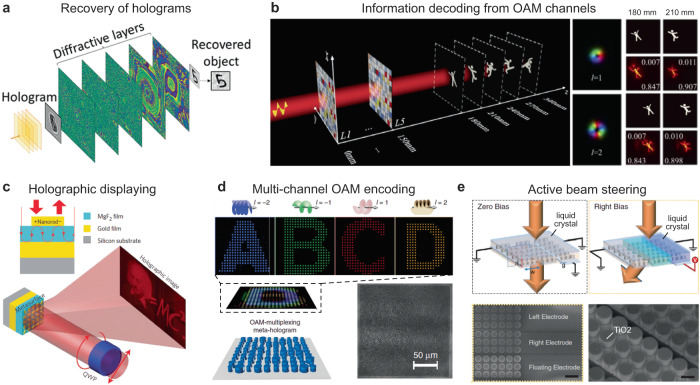
Diffractive networks and metasurfaces for all-optical spatial processing of light. All optical image recovery by diffractive networks from (**a**) computer-generated holograms^92^ and (**b**) OAM information channels^95^. Metasurfaces for (**c**) single channel^98^ and (**d**) OAM-enabled multichannel holography^99^. (**e**) Active electrically tuned spatial modulation by metasurfaces in liquid crystal cells^100^. **a** This is adapted with permission from ref. ^92^ by ACS. **b** This is adapted with permission from ref. ^95^ by © The Optical Society. **c** This is adapted with permission from ref. ^98^ by Springer Nature. **d** This is adapted with permission from ref. ^99^ by Springer Nature. **e** This is adapted with permission from ref. ^100^ by AAAS.

The ability of diffractive networks to process spatially encoded optical information can also enable hologram reconstruction and all-optical recovery of quantitative phase information (Fig. 5a)^67,92^. Quantitative phase imaging (QPI) is a powerful label-free imaging method for imaging low-contrast samples with weak scattering by visualizing the optical path length information of specimens^93^. By converting the input phase information of a scene into intensity variations at the output plane, diffractive networks have achieved QPI in a compact diffractive design that axially spans ~200–300 *λ*^93^. In contrast to digital approaches that retrieve holographic images from captured intensity images using digital phase recovery algorithms, these passive free-space processors enable all-optical reconstruction of holographic images, achieving superb image quality and high diffraction efficiency that can be generalized to classes of objects different from the training datasets^92,93^.

Diffractive networks also allow the separation of spatially multiplexed information encoded in different OAM channels that profoundly impact future telecommunication technologies^94^. Upon illumination by vortex beams that carry OAM, the engineered surfaces produce a selective set of images at designed spatial positions tailored by the orbital quantum number (*l*) (Fig. 5b)^95^. Metasurface-based processors for manipulating OAM with polarization and phase modulation have also been demonstrated^96^. Although existing demonstrations focus largely on terahertz waves and longer wavelengths, expanding to the visible ranges is expected with the developments in nanofabrication methods. We expect this technology to enable advances in holography and storage with larger scalability and information density.

Metasurface spatial processors have been demonstrated as single-layer devices, for example, to generate holograms based on computer-generated phase and amplitude profiles (Fig. 5c)^97,98^. By sampling with a 2D Dirac comb function in the spatial frequency-domain, a metasurface allows the multiplexing of multiple holographic images in different OAM channels by the same design (Fig. 5d)^99^. With integration with actively tunable optical materials, metasurfaces also allow beam steering and focusing of free-space light tunable by external stimuli such as mechanical deformation and electrical biases^100–103^. In particular, a one-dimensional SLM has been realized by liquid crystals (Fig. 5e)^100^. The ability to reconfigure metasurfaces has contributed to adaptive holograms where the image can be tailored by chemical^104^ and mechanical stimuli^105^. Some of these wavefront modulation and beam steering applications shown in, e.g., Fig. 5c–e are relatively limited in their computational capabilities since they perform a restricted form of wave transformation with limited input-output representations as opposed to implementing a function that can accurately transform infinitely many optical inputs to the desired representations at the output aperture (such as, e.g., Fig. 5a). With further advances in 3D nanofabrication techniques, we expect the realization of multi-layer metasurface networks to enable powerful adaptive free-space spatial processing and function representation capabilities critical for next-generation information processing and VR/AR applications.

### Optical processing of spectral and temporal information

Diffractive surfaces also present powerful tools for processing the spectral and temporal information of light. One area of application that has long exploited diffractive surfaces to manipulate the temporal profile of light is the shaping of broadband optical pulses in a vast range of applications, e.g., lightwave communications, biomedical imaging, and sensing. Typically, diffractive pulse shaping systems^106^ consist of a dynamic/passive diffractive modulation surface sandwiched between two Fourier transform lenses (or concave mirrors)^107^. In this conventional setup of optical pulse shaping, using metasurfaces with advanced meta-units, e.g., Si nanopillars on a fused-silica substrate, brings additional degrees of freedom and capabilities leading to, e.g., ultra-wideband operation^108^. However, one particular drawback associated with this standard pulse shaping architecture is that it often results in bulky optical systems due to the use of lenses in paraxial regime (satisfying the condition for Fourier transformation) in addition to other components such as gratings and mirrors^109^. Moreover, while high-quality lenses, mirrors, and gratings are widely available for applications in certain parts of the electromagnetic spectrum, e.g., visible range, for optical pulses occupying some other parts of the electromagnetic spectrum, e.g., THz, the quality of these components is often inadequate. Deep learning-based design of diffractive optical processors, on the other hand, offers lens-free, data-driven, and compact alternatives for optical pulse shaping. Experimental demonstration of a compact, lensless pulse shaping platform that relies on 3D-printed dielectric diffractive surfaces manipulating an input broadband THz pulse has been reported^18^.

In the context of broadband light processing, task-specific dielectric diffractive surfaces structured using deep-learning can also provide solutions to various challenging inverse design problems, including single/multi-passband spectral filters and spatially-controlled wavelength-demultiplexing^16^. The advantages of a deep-learning-based task-specific design of diffractive optical processors can be further exploited by replacing the dielectric modulation surfaces with metamaterial-based solutions developed for applications in e.g., achromatic flat optics^40,41^. The additional degrees of freedom provided by the sophisticated meta-units, e.g., GaN nanopillars Al_2_O_3_ substrate, can enhance the broadband light processing capabilities of diffractive processors by providing e.g., simultaneous tuning of modulation response and dispersion characteristics of individual meta-units.

### Optical processing of polarization information

**Fig. 6:**
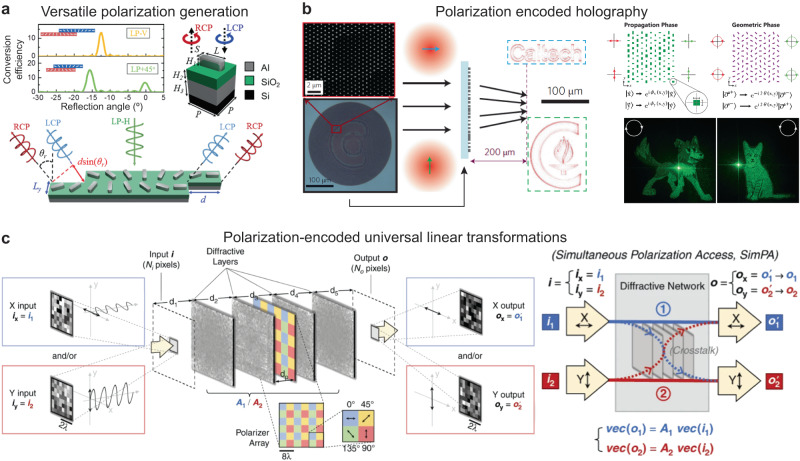
Free-space polarization processors based on diffractive networks and metasurfaces. **a** Metasurfaces for versatile polarization generation by nanorod units^112^. **b** Polarization-dependent holographic images using anisotropic meta-units^36,113^. **c** Diffractive networks enabled multichannel universal linear transformation by polarization multiplexing using a polarizer array^14^. **a** This is adapted with permission from ref. ^112^ by ACS. **b** This is adapted with permission from ref. ^36^ by Springer Nature and ref. ^113^ from APS. **c** This is adapted with permission from ref. ^14^ by CC BY 4.0.

Since its invention, metasurfaces have been widely used for the manipulation of polarization states using their anisotropic subwavelength units that exhibit polarization-selective optical responses^23,30,38,49–51,53,110,111^. Based on their in-plane orientations (*φ*), P-B geometric phase units such as nanorods generate opposite phase changes (±2*φ*) on left- and right-hand circularly-polarized light, respectively (Fig. 6a)^112^. Therefore, a metasurface with a linear phase gradient can separate a linearly polarized incident beam into two circularly-polarized beams in the opposite direction. A combination of two P-B supercells of meta-deflectors with opposite phase gradients at a subwavelength scale can generate all polarization states by tuning the shift distances (Fig. 6a). Polarization-sensitive metasurfaces can also produce holographic images encoded in different cross-polarization channels (Fig. 6b)^36,113^. Existing metasurfaces have demonstrated polarization processing by single-layer designs that typically accept only plane-wave inputs. Although multilayered metasurface polarization converters have been demonstrated in the literature^114,115^, these reports are based on periodic lattices and form single-pixel polarization processors. Metasurface-based networks for versatile polarization processing are still lacking but important for high-throughput information processing systems. This leaves significant room for improvement: a multiscale design strategy that concurrently optimizes the unit-level designs and the global structures of metasurfaces can provide unprecedented possibilities for polarization processing.

We want to point out that diffractive surfaces consisting of isotropic materials and unit cell designs can also realize polarization processing. Combined with a polarizer array, an all-optically polarization-multiplexed diffractive processor^14^ has been demonstrated to conduct multiple linear transformations by a series of isotropic diffractive layers (Fig. 6c). Fundamentally, this diffractive processor (composed of isotropic dielectric diffractive layers) gained its polarization-processing capability by spatial modulation of polarized beams generated by the polarizer array, which acts as a polarization seed within the free-space diffractive processor. Designed by a deep-learning training procedure, this free-space platform can perform concurrently multiple complex-valued matrix-vector operations through different polarization-encoded channels as long as the total number (*N*) of diffractive neurons satisfies *N* ≥ *N*_*i*_*N*_*o*_*N*_*p*_, where *N*_*i*_, *N*_*o*_ are the number of pixels at the input and output fields, and *N*_*p*_ is the number of transformations encoded as combinations of input-output polarizations. This unique design strategy that achieves universal polarization transformations^15^ with global spatial modulation at the wavelength scale can also be combined with the unit-level polarization-tuning of metasurfaces to further enhance their processing capabilities.

## Hybrid systems: Integration of free-space optical processors with electronic computing

**Fig. 7:**
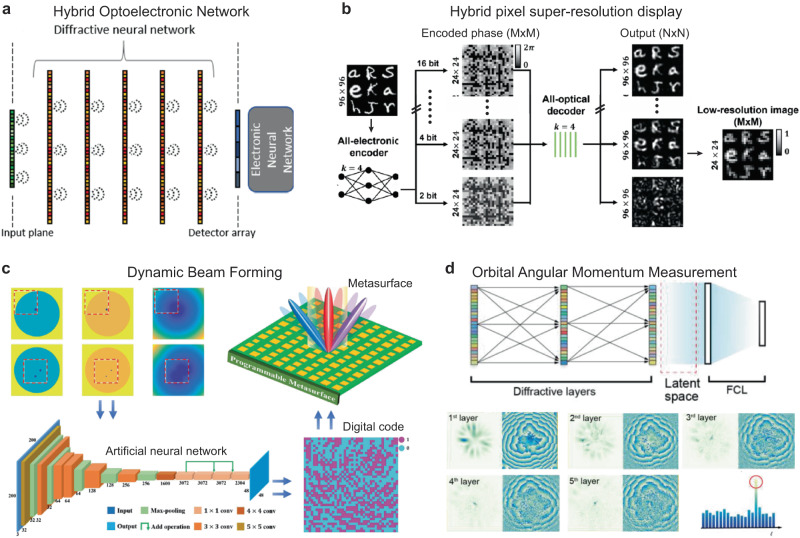
Hybrid optical-electronic networks for intelligent free-space processing. **a** Hybrid optoelectronic network for classification^66^. **b** Digital encoder enables pixel super-resolution displaying by diffractive networks^91^. **c** Deep-learning design of digital code for dynamic beamforming on a reconfigurable metasurface^120^. **d** Robust, high-accuracy OAM measurement by a hybrid network^123^. **a** This is adapted with permission from ref. ^66^ by CC BY 4.0. **b** This is adapted with permission from ref. ^91^ by AAAS. **c** This is adapted with permission from ref. ^120^ by IEEE. **d** This is adapted with permission from ref. ^123^ by CC BY 4.0.

Free-space processors that integrate both optical and digital neural networks^62,66,102,116^ can perform certain tasks with performances not possible by purely digital or optical components alone (Fig. 7a). This hybrid strategy has also been applied to integrated photonic systems^117,118^. For pixel super-resolution, which we have discussed in 2.3, for example, the digital CNN that encodes the high-resolution image into low-resolution SLM phase patterns not only serves as the essential interface to wavefront modulators but also reduces the burden of data transmission and storage (Fig. 7b)^91^. Hybrid networks have also realized machine vision using a single-pixel detector based on spectral encoding^119^. The diffractive network processes the spatial features of an input object illuminated by a broadband source and encodes the information into the power spectrum of the diffracted light that is processed by the free-space diffractive processor that is optimized using deep-learning to communicate with a single-pixel at its output. In addition to the classification of input objects based solely on the spectrum detected at the single-pixel output aperture, a fully-connected digital neural network can also reconstruct an image of the input object from the spectrally encoded classification signal detected by the single-pixel^119^.

We want to note that a single-step joint training of the optical and electronic parts often stagnates at a local minimum with undesirable performance. Instead, improved inference performance can be obtained from a two-step method that (1) trains the optical layers with additional virtual optical layers that act as a placeholder for the electronic network and then (2) jointly trains the untrained digital layers cascaded with the partially-trained optical front-end. This two-step training strategy achieves improved performance by providing a better initial condition for the diffractive network. Overall, this two-step transfer-learning-like training procedure can effectively augment the capabilities of a trained model and facilitates the integration of optical and electronic networks. The hybrid approach is also critical for increasing the depth of metasurface-based free-space processors^62^, which have been limited to a single optical layer by current limitations in nanofabrication. The depth advantages of optical and hybrid networks will be further discussed in Section “Tunability and reconfigurability”.

Digital deep-learning methods can also guide the structural design and reconfiguration of diffractive and metasurface devices^120^. Reconfigurable phase layers and metasurfaces can be realized by SLMs (in the visible) and programmable optoelectronic units (GHz range) (Fig. 7c)^120,121^. Hybrid neural networks also provide powerful tools for the recovery and measurement of phase-encoded information. For example, phase-encoded images formed by overlapping two phase images of MNIST numbers can be classified by a diffractive processor, and each digit can be accurately reconstructed by a digital network^122^. The optical network compresses the size of the input phase image to reduce the number of pixels by 20–65 times, which facilitates the reconstruction of both images regardless of the phase ambiguity. The ability to process phase information also allows the hybrid systems to manipulate OAM beams, which form a promising technology for next-generation information processing and telecommunication (Fig. 7d). Remarkably, this diffractive network integrated with a single fully-connected layer can achieve high calculation speed and energy efficiency where 99.98% of computations are performed optically. The OAM reconstruction achieved an extraordinary accuracy with a mean-squared error (MSE) of 10^−5^–10^−3^ ^123^. The compact system (footprint ~200*λ*) can achieve high robustness to the atmospheric turbulence and the spatial displacements of OAM beams, including transverse and angular shifts. Recently, a demonstration of electrically tuned metasurfaces can generate the OAM beam with dynamically-tunable topological charges^124^. We believe that the integration of digital neural networks with these reconfigurable (ideally jointly-trained) metasurfaces can contribute to a broad range of futuristic information technologies.

## Grand challenges in free-space optical computing and performance limitations

Various practical applications of free-space optical systems, such as imaging and computing, require tackling various challenges in device fabrication and performance. Diffraction efficiency, in particular, is critical for enabling energy-efficient technologies ranging from QPI and microscopy to holographic displays. Emerging innovations in the design methods and optical materials that can improve output diffraction efficiencies will be discussed in Section “Diffraction efficiency and power requirements”. The ability to tune and reconfigure free-space devices during their operation is also critical for various applications, including telecommunications (e.g., active beamforming) and imaging. To discuss these challenges, we will cover the critical problems and emerging opportunities in realizing tunable and reconfigurable diffractive and metasurface-based networks in Section “Tunability and reconfigurability”.

Furthermore, the nanofabrication techniques needed for building these 3D optical networks will be discussed in Section “Fabrication complexity and 3D alignment requirements”. Section “Computation speed, parallelism, and scalability” will analyze the strategies to achieve fast, parallel free-space processors with the scalability that is essential in modern computing platforms for solving complex problems. Lastly, for free-space processors to become a game-changer for the next-generation of computing technologies, they need to achieve competitive computation accuracy and precision compared to their electronic counterparts in the computational tasks they are designed for. Some of the potential techniques to improve the accuracy of diffractive and metasurface-based networks for computation and inference tasks will be discussed in Section “Computation accuracy and inference capability”.

### Diffraction efficiency and power requirements

The overall diffraction efficiency of metasurface systems, defined as the percentage of the optical power detected at the targeted sensor pixels with respect to the total input power, has long been a limiting factor for achieving competitive performance in imaging and display technology^125^. Plasmonic metasurfaces operating in the transmission mode, in particular, often exhibit low diffraction efficiencies that prohibit their applications in imaging and spectroscopy. Plasmonic meta-units exhibit non-radiative losses due to their materials properties and suffer from radiative modes that direct ballistic photons to undesirable targets. While the search for low-loss plasmonic materials in the visible and near-infrared range is still an ongoing research effort^56,126–128^, designs of metasurfaces that operate in reflection mode have realized a diffraction efficiency of >80% for holography^98^ and beam steering^129^ by using metal-insulator-metal structures.

All-dielectric metasurfaces can realize higher power efficiencies for transmission applications using materials such as TiO_2_ for the visible range^38^ and Si for the near-infrared range^64^. By tailoring their size and shape, the meta-units can exhibit destructive interference of electrical and magnetic dipole modes^130^ in reflection to suppress reflection losses and enhance transmission efficiency^125^. Ideal designs of dielectric P-B phase elements (such as nanofins) can function as perfect half-wave plates that shift the phase of the incident light polarized along the long axis of the meta-units by π with respect to the light polarized along their short axis. Experimentally, the focusing efficiency of such P-B devices can achieve ~86% at 405 nm^38^. In comparison, polarization-insensitive metasurfaces consisting of cylindrical dielectric posts that tailor the wavefront phase by their diameters have reported diffraction efficiencies higher than 90%^131,132^. Furthermore, broadband, polarization-insensitive metasurfaces have recently achieved a diffraction efficiency of 90% in the 450–700 nm wavelength range by dispersion-engineered meta-units^133^.

This relatively high efficiency of dielectric metasurfaces comes at the cost of difficult fabrication, which will be discussed in Section “Fabrication complexity and 3D alignment requirements”. Briefly, commonly used dielectric materials such as TiO_2_ and Si are produced by slow and costly methods such as ALD and CVD. Realization of multi-layer metasurface designs by these high-temperature processes, which preclude organic layer-to-layer spacers, is also difficult. Therefore, we believe that there are still tremendous opportunities in the discovery of low-loss, high-index optical materials that allow simpler fabrication. The dielectric nanoposts with small diameters typically have a very high aspect ratio that makes their accurate nanofabrication challenging, and therefore, the experimental diffraction efficiency can be compromised due to fabrication imperfections.

Diffractive networks and dielectric-based optical processors composed of *λ*/2 lateral feature sizes (Section “Diffractive surfaces”) exhibit power efficiencies that largely depend on their training loss function and design process. For example, diffractive network training for all-optical image classification using MSE loss promotes higher signal contrast and better power efficiency (routing e.g., 25.07% of the output photons to the correct photodetector assigned to the correct label for MNIST image data) while the cross-entropy loss favors a significantly better classification performance with a compromise in the diffraction efficiency (by e.g., ~10-fold)^66^. In general, the output diffraction efficiency of a multilayer diffractive network shows a depth advantage where an increase in the number of diffractive surfaces/layers improves its overall power efficiency because of the additional degrees of freedom in the system^66,134^. In these cascaded optical processor designs, the diffractive efficiency at the output plane can be further improved by a compromise in the performance of the network, such as imaging contrast, signal-to-noise ratio, and test object classification accuracy. This trade-off between output diffraction efficiency and performance can be engineered readily by the choice of loss functions and the level of diffraction efficiency penalty^18,66,92,93^. For example, for a diffractive processor design that performed all-optical QPI through the conversion of phase-only signals into quantitative intensity patterns^93^, an increased output diffraction efficiency of >11% was achieved by increasing the weight of the diffraction efficiency related penalty term in the loss function at the cost of a slight decrease in the image quality (i.e., a structural similarity index reduction of <0.06). A similar trade-off was observed in the all-optical reconstruction of holograms^67,92^, where the diffraction efficiency of the reconstructed holographic images at the output plane was increased to >26% by increasing the weight of the diffraction efficiency penalty term in the training loss function, which resulted in a tolerable compromise in the reconstruction quality.

In experimental validation, however, material absorption can lead to a larger loss with an increasing number of successive layers (or thicker monolithic designs) if a low-loss material for the fabrication of the 3D diffractive processor is not available for the wavelength range of operation. In the network training process, the lossy diffractive layers can be modeled as complex modulators (Section “Diffractive surfaces”) using the measured extinction coefficient of the material so that the design parameters (e.g., the number of layers, layer-to-layer separation, etc.) can be optimized during the training. For the visible part of the electromagnetic spectrum, this poses less of a challenge since there are various low-loss optical materials (e.g., glasses, polymers) that can be engineered at the wavelength scale in 3D^135–137^. Materials such as TiO_2_ and Si are also suitable for building low-loss diffractive layers in the visible and near-infrared wavelength ranges, respectively.

### Tunability and reconfigurability

**Fig. 8:**
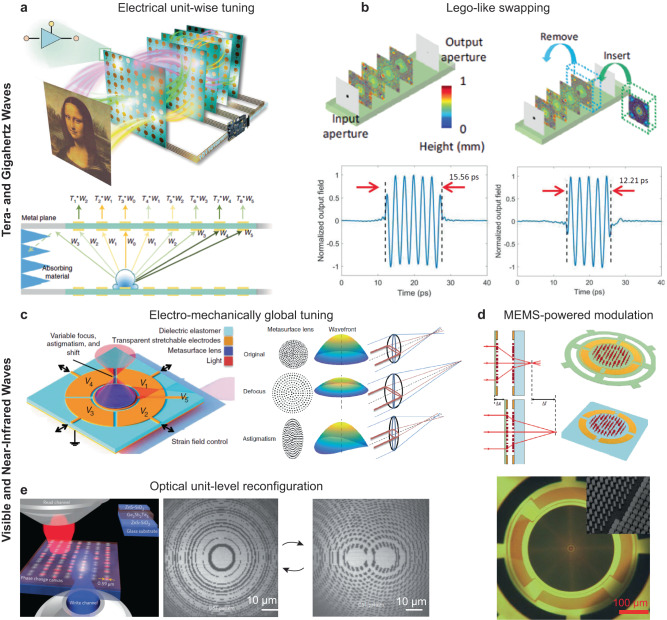
Tunable and reconfigurable responses from metasurfaces and diffractive networks. Tuning of device responses by (**a**) Lego-like swapping of the layer designs^18^, (**b**) electromechanical modulation that deforms the metasurface substrate^101^, (**c**) MEMS systems that adjust distances between two metasurfaces^102^. Unit-level metasurface reconfiguration by (**d**) electrical tuning of field-programmable gate arrays^121^ and (**e**) reversible laser writing on phase-change materials^144^. **a** This is adapted with permission from ref. ^18^ by Springer Nature. **b** This is adapted with permission from ref. ^101^ by AAAS. **c**–**e** These are adapted with permission from ref. ^102^, ref. ^121^, and ref. ^144^, respectively, by Springer Nature.

The ability to fine-tune the design of free-space optical processors is critical for adjusting their performance based on the task, which is especially important when more than one metric needs to be optimized concurrently^138,139^. In a typical example of an image classification problem, the main objective to optimize is the classification accuracy, but the power efficiency and signal contrast are also important in practical applications. Therefore, on-demand tuning of the network after the fabrication is critical for optimizing the performance of these free-space processors based on the need of the specific tasks. Diffractive free-space processors consisting of transmissive layers also allow Lego-like swapping of layers to tune their output function (Fig. 8a)^18^. For example, based on a transfer learning approach, some existing layers in a pre-trained diffractive network can be physically replaced with newly trained and fabricated transmissive layers to enable the on-demand synthesis of new pulses with desired pulse shapes and durations. The connectivity of a diffractive network, which can be tuned by the layer-to-layer distance Δz, is another important parameter that determines the output of the diffractive optical processor^16^. For terahertz pulse shaping, for example, the same set of diffractive layers produced a tunable center frequency from 0.349 THz to 0.399 THz by physically reducing Δz from 30 mm to 25 mm^18^. To apply this Δz tuning of diffractive and metasurface layers that operate at the visible part of the spectrum, we expect MEMS-based systems^102,140^ to become useful for the precise control over Δz at micrometer length scales. Alternatively, the effective optical path length between the passive layers can be tailored by modulating the background refractive index without changing the physical distances between the layers. This index-tunability can be realized by a range of materials systems, including liquid crystals^100^, electro-optic materials^141–143^, and phase-change materials^144^.

The tunability of metasurfaces is also important for achieving adaptive responses in applications ranging from imaging to spectroscopy. Mechanical tuning, for example, is a common approach for realizing active metasurfaces with tunable global optical responses^103,105^. By patterning meta-units on a flexible electroactive substrate, the focal point of a metalens can be shifted and stretched upon applying electrical biases, which adjusts the spacings (*a*_0_) between the meta-units (Fig. 8b)^101^. Microelectromechanical systems (MEMS) that adjust the distance between two metasurfaces can also tailor the output plane (Fig. 8c)^102^. The ability to shift and reshape the foci of metalenses is critical for building compact microscopes and miniaturized cameras. These mechanical systems based on adjusting lattice constants can also modulate the spectral responses of the metasurfaces^145,146^. By adjusting the spacing between the meta-units, the resonance of a plasmonic metasurface can be tuned over a wide wavelength range (e.g., 500–600 nm). In general, mechanical modulation is slow (e.g., with a response time of tens of ms)^101^ and limited to changing the global structures of metasurfaces but can not access and individually change their degrees of freedom. Integration with emerging 2D materials such as graphene^147,148^ and black phosphorous^149^ is also promising to enable gate-tunability within metasurface networks. In addition, metasurfaces with a silicon-on-lithium niobate architecture demonstrated wavefront shaping and modulation based on electro-optic tuning^150^.

In addition to tunability (fine-tuning the function of an optical processor), the reconfigurability of optical processors allows these devices to evolve and adapt to different tasks to achieve optimal performance in a dynamic environment. For diffractive free-space processors, fast reconfiguration at the single-neuron level can further enable in-situ network training, where the diffractive devices are tailored in real-time based on their measured responses^27^. This training strategy that uses experimental results instead of simulated responses can not only realize accurate evaluation of the optical processor performance but also reduce the training time for dynamically changing tasks whose simulation is computationally expensive. Gate-tunable plasmonic units were realized by graphene-gold resonators with a single-unit phase tunability of 0–1.28π in the mid-infrared range and were used for achieving active beam steering^147^. A fully-programmable metasurface network was also demonstrated for GHz wave applications using electrical field-programmable gate arrays (Fig. 8d)^121^. These GHz wave processors can be reconfigured to perform a range of tasks ranging from matrix inversion^151^ to image classification and information encoding/decoding^152^. The ability to conduct fully-programmable field synthesis is also valuable for beamforming and holographic image projection applications^153^. For visible and near-infrared ranges, however, unit-level adjustment of the diffractive surfaces and metasurfaces is more challenging because the feature sizes are significantly smaller. Unit-level reconfiguration has been realized by focused laser writing and erasing of phase-change materials^144,154^ such as Ge_2_Sb_2_Te_5_ (Fig. 8e). This approach, however, requires sophisticated instrumentation for the active modulation and can not apply to multi-layer processors with current technology.

Overall, reconfigurable diffractive and metasurface processors are expected to have more complex structures and fewer materials choices that are harder to fabricate than their static counterparts. For visible-range applications, in particular, the development of fully-dynamic nanoscale meta-units with comparable fine features as static meta-units is extremely challenging with the current nanofabrication techniques. However, reconfigurable free-space devices with competitive performances can be realized with a new paradigm of hybrid network structure consisting of (1) fixed diffractive and metasurface layers with carefully engineered subwavelength features that provide the fine control over optical wavefront, (2) tunable optical superstructures with micron-scale features sizes for introducing tunable and reconfigurable optical responses to the system, and (3) digital networks for the pre- and post-processing of the input/output. We expect this hybrid framework to become the next-generation of high-performance reconfigurable free-space processors that can be integrated with portable devices.

### Fabrication complexity and 3D alignment requirements

Precise fabrication of diffractive free-space processors is important for ensuring the accuracy of these analog computing platforms, which do not have the same error-correction ability as digital computing. For these optical computing systems, in particular, the misalignments between the diffractive or metasurface layers will introduce potential systematic errors to the computing results and should be ideally limited to a size comparable to the illumination wavelength. The ability to mitigate these misalignment errors is essential for fully harnessing the computing capabilities of multilayer 3D diffractive networks. Therefore, the fabrication of diffractive free-space processors that operate at visible wavelengths requires the ability to create layers of nanoscale units with accurate phase modulation using e.g., spatial engineering of the refractive index and/or thickness of the material. For instance, 3D printing methods based on two-photon polymerization of photosensitive materials^45,46^ are promising techniques for achieving subwavelength feature resolution using tightly focused laser beams (Supplementary Fig. S2a). Commercial solutions such as the Quantum X developed by Nanoscribe^135,136^ and MicroFAB-3D developed by Microlight3D^43^ have demonstrated the printing of sophisticated photonic structures such as photonic crystals^155^ with a resolution below 200 nm. Implosion lithography^137^ can further push the resolution limit down to 50 nm by printing a magnified version of the target structure in a hydrogel and shrinking the entire structure by dehydration (Supplementary Fig. S2b). One of the major challenges in creating visible-range diffractive free-space processors consisting of multiple thickness-tuned layers is their mechanical robustness. Although structural supports are added between the diffractive layers to be printed/fabricated in the same fabrication session, the layers during the curing process can undergo deformations that can produce inaccurate layer-to-layer distances and degrade the performance of the optical processor. Therefore, monolithic architectures would be preferred for building robust, miniaturized diffractive free-space processors. For example, laser writing techniques^48^ can generate index-tuned diffractive layers in a single dielectric volume to achieve high mechanical robustness. A current limitation of this technique is that the index-tuning by exposure time may have limited spatial resolution and index-tuning range. Therefore, the discovery of novel material platforms that allow index-tuning is critical for building robust, monolithic diffractive optical processors.

On the other hand, optical metasurfaces are typically fabricated by an electron beam lithography process consisting of steps including electron beam writing, deposition, and etching (Supplementary Fig. S2c)^65^. In a typical fabrication process for a visible-range metasurface made of TiO_2_ (a high-index, low-loss dielectric material), the metasurface is fabricated by (1) electron beam writing of the design on the resist, (2) ALD-based fabrication of the TiO_2_ with excess thickness, and (3) etching of the excess TiO_2_ and removal of the electron beam resist to obtain the final metasurface pattern^38,65^. Si- or GaN-metasurfaces can be fabricated by similar processes where an etch mask with the metasurface design is produced by electron beam lithography and then the pattern is transferred to the Si or GaN film by anisotropic reactive-ion etching^36,40^.

Current fabrication methods for free-space metasurface processors, however, can only realize single-layer designs^42,62^ or multilayer designs that do not require accurate (<*λ*/2) layer-to-layer alignment^64,156–158^. An alignment technique between different fabrication layers is still lacking and this feature is essential for achieving multilayer metasurface processors for performing e.g., universal linear transformations in a transmissive geometry between a diffraction-limited input field and output. Also, interlayer structural supports are needed to construct multilayer metasurface-based optical processors. Unlike their THz counterparts that can be assembled from free-standing layers in the air, visible-range metasurfaces must be fabricated on robust, smooth substrate materials. Therefore, dielectric filler materials with high transparency and mechanical stability would be preferred spacers that separate the metasurface layers. The major challenge is that deposition methods such as ALD and PECVD for high-quality transparent materials are slow (<5 nm/min for ALD of Al_2_O_3_^159^ and 167 nm/min for PECVD of SiO_2_ by SAMCO) and expensive. The residual stress accumulated during these high-temperature deposition processes also makes the film vulnerable to cracking and fracture at thicknesses larger than 15 µm^160^, which restricts the designs of metasurface processors that can be realized. The poor thermal conductivity of common dielectric materials also produces cooling issues in dry etching processes and prohibits the directional etching required for producing high-aspect ratio dielectric meta-units^161^. We expect the development of novel dielectric materials and nanoscale 3D alignment methods to be crucial for realizing metasurface networks formed by successive layers of jointly-optimized meta-layers.

In general, nanofabrication methods that allow scalable, low-cost production of metasurface networks are essential for the wide-scale use of such optical processors. One promising approach to realizing large-scale parallel nanofabrication is photolithography which can replicate the metasurface designs defined on a photomask on transparent wafers (Supplementary Fig. S2d)^162^. Using a 193-nm light source and liquid-immersion technology, the state-of-the-art deep-ultraviolet (DUV) lithography processes can easily produce meta-units below 40 nm^163^. Inverse design of photomasks using sub-resolution assist features can further improve the quality of the fine features^164^. The main disadvantage of DUV photolithography is its extremely high instrumental cost. In contrast, soft lithography methods such as nanoimprinting^165^ and solvent-assisted nanoscale embossing^166^ can produce metasurfaces with features below 60 nm using benchtop (Supplementary Fig. S2e) and are suitable for usage in labs and small-scale manufacturing. In a typical soft lithography process, an elastomeric stamp is obtained by molding from a template with the metasurface design and brought into conformal contact with a photoresist-coated substrate. Via mechanical pressure or solvent interactions, the metasurface designs are formed on the substrate and subsequent pattern transfer steps such as deposition and etching are used to produce the metasurface devices.

Besides direct improvements in instrumentation and materials, the challenges in nanofabrication of diffractive and metasurface-based free-space optical processors can be resolved in the design process by considering the imperfections in fabrication. Previously, diffractive networks that are invariant to the scale-, shift, and rotation of input images have been demonstrated by introducing these variations in the training stage^167^. An extension of this approach can tremendously improve the robustness of the free-space optical processor toward the unavoidable misalignments between the layers or other imperfections by including them as random parameters in the training process^82,90,168^. We expect this ‘vaccination’ strategy to apply to a wide range of imperfections in fabrication and experimental systems (such as inaccuracy in phase and amplitude modulation) and can be customized based on the limitations of the selected fabrication method. Furthermore, in contrast to the designs that use analytic wave-optics approaches, the deep-learning-based diffractive and metasurfaces-based optical processors that are trained using data can be designed based on an incomplete phase coverage (less than 2π, that can be selected on demand) to accommodate materials-related refractive index limitations and phase restrictions to enable simpler fabrication methods. All in all, deep-learning-enabled designs of diffractive and meta-material based optical processors would be able to better handle some of these experimental restrictions due to fabrication imperfections and limitations in e.g., resolution, feature size, depth, refractive index, and mechanical misalignments. There is still considerable work needed for building cascaded free-space optical processors that can operate at IR and visible wavelengths at diffraction-limited SBP, in order to fully utilize the density of information encoding in free-space.

### Computation speed, parallelism, and scalability

Free-space optical processors present a unique advantage since their computation is completed as the input light propagates through the optical processor and its volume/layers. While electronic processors and integrated photonics-based processors also utilize the speed of electromagnetic wave propagation, the free-space processors *complete* all the calculations and inference tasks with a single pass of wave propagation, without any digital storage/transmission or pre-processing of information. Therefore, the computing speed of diffractive and metasurface systems for the same task can be increased drastically by scaling the system down to the nanoscale feature sizes, operating at visible or IR wavelengths, with a total axial thickness of e.g., ~100–200*λ*. To manipulate this wavelength range, however, the diffractive features used in free-space optical processors need to have dimensions of >100 nm, significantly larger than the state-of-the-art transistors on chips made by the 3-nm process. In addition, the size of free-space optical processors that can be accurately modeled and designed (through e.g., a deep-learning-based training process) is still limited by the speed of digital computers and their memory restrictions. Unlike CPUs and GPUs that can be assembled into clusters to solve advanced computation problems, a versatile strategy to assemble diffractive networks into a large-scale cluster of free-space optical processors is still lacking, but highly desired to fully utilize their advantages in parallelism. Therefore, the ability to construct low-loss, large-scale networks of diffractive optical processors is thus critical for building fast, free-space computing platforms with significantly lower power consumption.

One promising strategy to achieve a high density of processing units in free-space optical processing is to use a *diffractive volume* where every discrete cell in the 3D space is spatially engineered to collectively process the input optical information. Such a volumetric optical processor can have a very large number of optimized diffractive features, e.g., >10^9^ within a compact material volume, which would normally be challenging for the multilayered designs to achieve even with nanoscale meta-units. Such a large density of optimized diffractive features within a 3D compact topology could be another unique aspect of free-space-based visual information processors and is hard to match with planar integrated photonic circuit-based designs. Such dense 3D lattices of diffractive units also have the potential to utilize evanescent waves (containing much larger spatial frequencies) to further increase the computation capability. However, the design of a diffractive volume (that can process evanescent waves) is challenging not only because a fast and accurate forward model that can quantify the scattering between these densely-packed units is lacking, but also due to the extreme difficulties in the fabrication of such monolithic free-space processors. For example, a nanophotonic design volume based on simulations by finite-difference frequency-domain calculations and fabrication by laser-tuning of refractive index was demonstrated, but the total volume is limited to 4*λ* × 4*λ* × 6*λ*^169^. This diffractive computing medium needs to scale up significantly using e.g., emerging nanofabrication^137^ and optical simulation methods^170^. For scaled-up designs, the optical simulation technique used by the forward model must be fast and accurate while allowing the computation of gradients essential for the deep-learning training process. Well-established numerical methods such as the finite-elements method^171^ and finite-difference time-domain^172^ exhibit high-accuracy, but can not meet the speed and gradient-computation requirements. One promising simulation method is MaxwellNet^170^, a physics-driven neural network that can solve for the optical responses of a diffractive volume. Using the residual of Maxwell’s equations as the losses, the method can be trained to quickly predict the phase and amplitude modulation of a structure given the spatial distribution of the material property.

Despite the challenges above, the diffractive and metasurfaces still have tremendous opportunities and advantages in information processing because of their unique operation. For example, the massive parallelism of optical systems allows the same free-space optical processor to perform multiple tasks simultaneously with the multiplexing of wavelength, polarization, and spin^14,17^. In particular, the subwavelength structures of meta-units provide additional structural degrees-of-freedoms that allow independent, pixel-level control over e.g., polarization^112,113^ and dispersion^173^. We believe that rationally-designed metasurface-based networks can form multispectral, polarization-multiplexed free-space processors that can process each channel independently while providing on-demand control of the cross-coupling among different channels—all within the diffraction limit of light and the corresponding SBP of the input and output apertures. In addition, free-space processors can be integrated directly into the light path (without the need for digitization, storage/transmission, and pre-processing of information) to perform tasks such as image reconstruction and class-selective imaging with zero time delay^28,90^. Therefore, we expect that free-space optics will form powerful task-specific processors to perform analog computation and imaging tasks at the speed of light.

Overall, diffractive surfaces and metasurfaces can potentially provide a scalable free-space platform for highly parallel processing of visual information that is difficult for integrated photonics-based systems to process directly, which are, by design, more suitable for processing already digitized information. For example, the 2D phase information of a scene/object can be directly processed by free-space-based diffractive processors without the need for pre-processing of information (such as phase retrieval and vectorization of the resulting phase image) which would normally be required for an integrated photonics-based processor before it can act on the phase image. Free-space optical processors, in contrast, can directly process 2D or 3D visual information and work with significantly larger inputs since their physical size linearly grows with the size of the input FOV. For inference tasks such as image recognition and classification, free-space optical processors based on diffractive and metasurface networks allow highly dense connectivity in 3D, which is harder to achieve in integrated photonics^174^. Although the number of trainable parameters of optical neural networks, as of today, is still significantly smaller than the state-of-the-art electronic deep neural networks^175^ (with, e.g., ~10^12^ parameters), free-space-based processors can still provide unique advantages due to their direct access to the optical information of input scenes/objects without the need for any digital cameras, image recording/transmission, or pre-processing of data, providing ultra-fast and power efficient visual information processors—potentially integrated with mobile devices.

### Computation accuracy and inference capability

For free-space diffractive and metasurface networks to play a key role in next-generation technology, they must reach a computation accuracy and precision comparable to their existing electronic counterparts in the target computational tasks. In digital electronic systems, the precision is determined by the number of discrete states implemented by binary logic units to approximate continuous variables^176^. Increasing the precision of analog devices, in general, requires significant advances in materials and fabrication techniques, where the cost can increase exponentially with increases in the desired precision^177,178^. In particular, free-space optical processors as analog optical information processing systems exhibit an explicit limit in their precision, practically limited by their design, fabrication, and measurement processes. For example, errors in diffractive computing can result from inaccuracy in the layer thicknesses or the topology of the fabricated subwavelength features due to imperfections of the 3D printing process. Misalignments between diffractive layers can also introduce nontrivial errors that lead to incorrect computational and inference outcomes^168^. Although statistical inference and computational imaging tasks usually do not require high precision^179^ and can tolerate some degrees of errors, other applications such as linear transformations^13^ can be much more sensitive. Some of these limitations in nanofabrication and misalignments can potentially be addressed by appropriate design and network training strategies that take these factors into account during the deep-learning-based optimization and training of the free-space processor (discussed in Section “Fabrication complexity and 3D alignment requirements”).

The computational performance of current deep neural networks systems can be understood by the universal approximation theorem^180^, which states that multi-layered networks with a sufficient number of hidden units and adequate nonlinear activation functions can approximate any continuous function mapping from closed, bounded space to another one with finite dimensions^180–182^. To achieve universal function approximation through free-space optical networks, however, materials and neuron designs that can achieve nonlinear optical responses would be needed^73^. Nonlinearity has been implemented in diffractive optical processors using a combination of photodetector/sensor arrays and dynamic electro-optic modulation devices^27,183^. However, this approach not only significantly increases the complexity of the devices but also introduces undesirable active power consumption to the system. The speed of operation will also be limited by the performance of the dynamic electro-optic modulation devices. Another interesting demonstration of nonlinearity using transitions between atomic states of ^85^Rb^184^, but the use of vulnerable atomic systems makes this system impractical in realistic devices. A practical material system that enables large nonlinearity and easy fabrication is still the main challenge in developing the next-generation of free-space machine-learning devices^185^. Besides the direct implementation of nonlinear activation functions, the inference capacity can also be improved by innovations in diffractive processor architecture and training strategies, including class-specific network training^68^ and ensemble learning^69^ as detailed in earlier sections. A disadvantage of both of these strategies (class-specific diffractive networks and ensemble learning approach) is that multiple diffractive networks need to operate together, requiring sophisticated 3D integration and optical alignment in free-space, further complicating the optical hardware. As an alternative approach, inspired by pixel super-resolution microscopy using object shifts^186^, time-lapse image classification using a single diffractive network was reported that utilized random or ordered lateral shifts of an object with respect to the input aperture to achieve ~62% inference accuracy for classifying CIFAR-10 test images using a single time-lapse diffractive optical network^187^.

## Conclusions

**Fig. 9:**
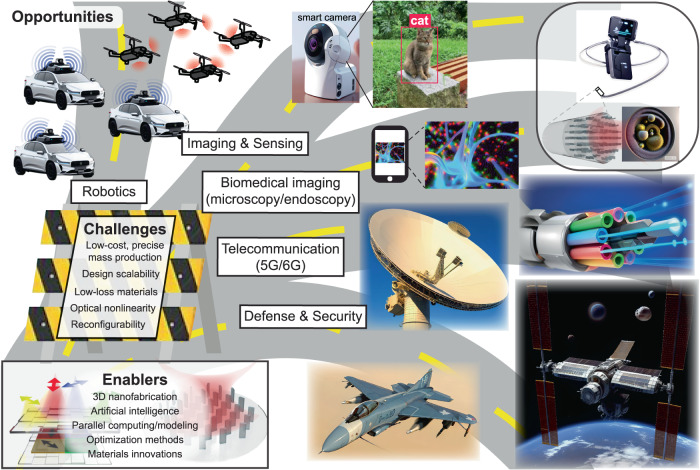
Roadmap and future outlook of diffractive surface- and metasurface-enabled computing platforms. These free-space optical processors can have transformative impact on various technologies ranging from robotics to biomedical imaging and telecommunications, among others.

In this Perspective, we discussed the recent advances in diffractive surface and metasurface designs as free-space optical processors and their implications and potential impact on future analog visual computing technologies (Fig. 9). Starting from their fundamental design and operation principles, we analyzed the capabilities and potentials of optical diffractive networks as a platform for statistical inference^26^ and universal linear transformations^13,14^. As discussed earlier, we expect major advances in the design and fabrication of diffractive and metasurface-based free-space optical processors to achieve more competitive performance in inference tasks, especially if the 3D alignment and assembly challenges in the fabrication of multi-layer designs can be overcome. We also reviewed some of the emerging approaches that can potentially overcome the practical challenges in implementing nonlinear activation functions^184^, a critical component for free-space optical processors to achieve enhanced performance.

As versatile analog computing platforms with machine-learning capabilities, these free-space optical processors form powerful *visual information processing* tools that can directly access and harness the spatial, spectral, polarization and amplitude/phase information of light waves. One critical application enabled by diffractive visual processors is all-optical QPI that can convert phase-encoded information of specimen into intensity patterns in a quantitative manner, performing all-optical phase recovery^93^, with various applications in biological imaging. Similarly, the ability to perform imaging through unknown, random diffusers using analog visual computing via spatially engineered diffractive layers^28^ or metasurfaces will open up new possibilities in, e.g., medical imaging, security screening and free-space communication. As another application, the class-specific imaging capability of diffractive visual processors further provides a novel solution to privacy protection problems that can be concerning during the deployment of data-driven imaging and surveillance technologies^90^. In addition to harnessing the spatial features of a scene, their inherent ability to process polarization^14,112,132^ and spectral/temporal^119,133,173,188^ information of light also makes these free-space optical networks promising for the development of next-generation sensing and telecommunication technologies. For example, both diffractive layers and metasurface-based designs possess the ability to manipulate structured light that carries OAM^63,95,123^, which is expected to be a core component of 6G communication technologies.

Finally, we have discussed in this Perspective some of the key metrics for evaluating the performance of free-space optical processors, including computation speed and scalability, power efficiency, and reconfigurability as well as the critical challenges that need to be addressed for achieving competitive scores on these metrics. In particular, the design and fabrication methods for massive-scale free-space optical processors with cascaded structures in 3D are still lacking. We envision that monolithic diffractive computing volumes^169^, as opposed to discrete engineered surfaces or layers, will form a powerful class of free-space computing platforms that can realize a significantly higher density of diffractive features in 3D; but such a capability needs a fast and accurate forward optical model in the design process, which will also need to carefully keep a record of the evanescent waves and their interactions with subwavelength structures within the spatially engineered volume. We expect that physics-informed deep neural networks^170^ trained to solve Maxwell’s equations rapidly would be one promising platform to be used in the forward model of such 3D computing platforms. These 3D designs will be capable of performing large-scale computing and optical information processing at low energy consumption and ideally below the diffraction limit of light. Another expected breakthrough in free-space optical computing would be the realization of dynamic tuning and on-demand reconfiguration of diffractive^18^ or metasurface-based^27,102,121,144,147^ optical processors. Rapid developments in various nanofabrication techniques, including nanolithography^162–165^ and 3D printing^43,45,46,136^, can accelerate the transition of dynamic diffractive and metasurface-based optical networks from GHz to visible frequencies. We also expect major advances in dynamic hybrid processors^62^ that combine fixed/static diffractive layers or metasurfaces with subwavelength features for fine control over the optical wavefront, adaptive optical superstructures for reconfigurability^27^, and digital neural networks that are jointly-optimized with the free-space optical processor for the pre- and post-processing^66,91,119,189^ of the input/output information.

### Supplementary information

Supplementary Information
