# Supplementary material for: Diffractive optical computing in free space
Source: Nat Commun. 2024 Feb 20;15:1525. doi: 10.1038/s41467-024-45982-w (PMC10879514; doi:10.1038/s41467-024-45982-w)
Supplement: Supplementary file 1 — Supplementary Information [file 41467_2024_45982_MOESM1_ESM.pdf]

SUPPORTING INFORMATION

Diffractive Optical Computing in Free Space

Jingtian Hu,[1,2,3] *Deniz Mengu,*[1,2,3] *Dimitrios C. Tzarouchis,*[4,5] *Brian Edwards,*[4] *Nader Engheta,*[4] *and Aydogan Ozcan**[,1,2,3]

[1]Electrical and Computer Engineering Department, University of California, Los Angeles, California 90095, United States.

[2]Bioengineering Department, University of California, Los Angeles, California 90095, United States.

[3]California NanoSystems Institute (CNSI), University of California, Los Angeles, California 90095, United States.

[4]Electrical and Systems Engineering, University of Pennsylvania, Philadelphia, Pennsylvania 19104, United States

[5]Meta Materials Inc., Athens, 15123, Greece

* ozcan@ucla.edu

Supplementary Discussion 1: Diffractive Surfaces and Metasurfaces

Structured surfaces, which include diffractive surfaces and metasurfaces, are the core building blocks of emerging free-space all-optical computing systems. These structured optical surfaces perform computation operations by controlling the degrees-of-freedoms of electromagnetic waves, including e.g., amplitude, phase, spectrum and polarization. This Box focuses on diffractive surfaces and metasurfaces based on phase modulation instead of their counterparts based on amplitude modulation, which generally suffer from limited diffraction efficiency and lower cascadability. **Figure S1a** shows the two most common types of diffractive surfaces that modulate the wavefront phase $\Delta\varphi(x, y)$ using dielectric unit cells with spatially varying height $h(x, y)$[1] or refractive index distribution $n(x, y)$[2] of the diffractive features, assuming a negligible material absorption. While operating in air ($n_{\text{medium}} = 1$), a single diffractive feature induces an optical path delay in the form of $\Delta\varphi = \frac{2\pi}{\lambda}h(n-1)$ and the overall 2D complex-valued transmission function of the diffractive surface is therefore defined as:

$$T(x,y) = exp\left(j\frac{2\pi}{\lambda}h(n-1)\right) \tag{1}$$

This diffractive layer will transform any incoming wavefront, $U_{\text{in}}(x, y)$, into an output wavefront described by $U_{out}(x,y) = T(x,y)U_{in}(x,y)$.[3]

Metasurfaces, on the other hand, tailor the optical wavefront with substructured unit cells with rationally-optimized subwavelength features (<λ/2). **Figure S1b** shows some of the frequently-used designs of the meta-units and their mechanism for manipulating the optical wavefront. Geometric phase units, for example, are anisotropic units with phase and polarization responses described by the Pancharatnam–Berry (P-B) principle.[4] Briefly, the meta-unit such as a dielectric nanofin[5] oriented at an arbitrary angle θ acts as a half-wave plate that induces an optical path delay of $\Delta\varphi = \pm 2\theta$ to the incident light with left- or right-hand circular polarization, respectively, while converting it to its orthogonal polarization state. Another class of metasurfaces consists of V-shaped plasmonic antennas that adjust the amount of phase delay by changing the length/width of their arms and the angle between them. The output polarization is identical to the input as long as the incident light is polarized linearly along the symmetric (Ŝ) or antisymmetric (Â) axes of the units. As another example, high-aspect-ratio dielectric nanopillars (height ~λ) can be used as meta-units when polarization-insensitive phase responses are desired. Full coverage of the 0-2π phase range can be achieved by adjusting the diameters of these dielectric posts.[6] Another emerging class of metasurfaces, i.e., nonlocal metasurfaces, uses symmetry-protected scattering from quasi-bound states in the continuum to realize ultra-sharp optical resonances with unique spectral responses.[7,8] The nonlocality allows the independent tuning of spectral and spatial selectivity to enable the implementation of wavefront-shaping and wavefront-selective devices.[9] Also, metagratings[10] with supercells discretized with a minimal number of 3 unit cells (scatterers) in each period can perform analog optical computation.[11]

Lastly, we want to point out that hybrid structured surfaces have also emerged. For example, a combination of diffractive surfaces and dielectric metasurfaces was realized by 3D printing[12] to achieve achromatic focusing (**Figure S1c**).[13] Similarly, a hybrid plasmonic-dielectric metasurface was also demonstrated for improving output diffraction efficiency (**Figure S1d**).[14]

Supplementary Figures

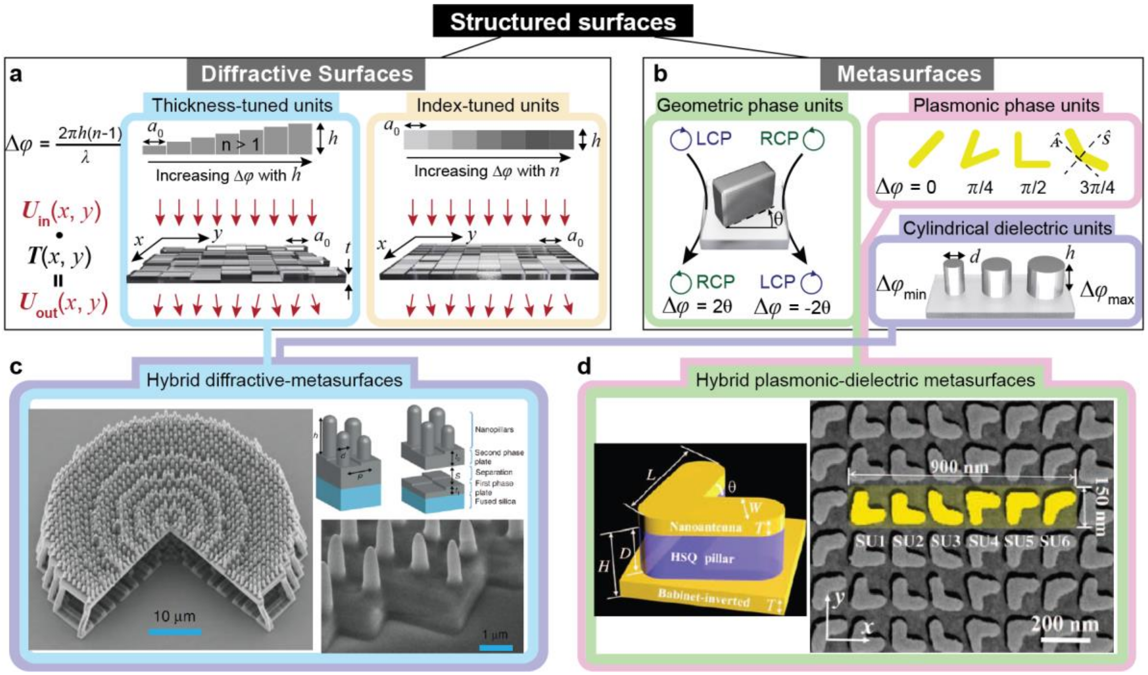

Figure S1: Types of structured surfaces for optical information processing. Schemes showing (**a**) diffractive surfaces based on thickness- and index-tuned units and (**b**) metasurfaces consisting of plasmonic or dielectric units. Examples of (**c**) hybrid diffractive-metasurfaces[13] and (**d**) hybrid plasmonic-dielectric metasurfaces.[14] Panel (c) is adapted with permission from REF.[13] under CC BY 4.0. Panel (d) is adapted with permission from REF.[14] by AAAS.

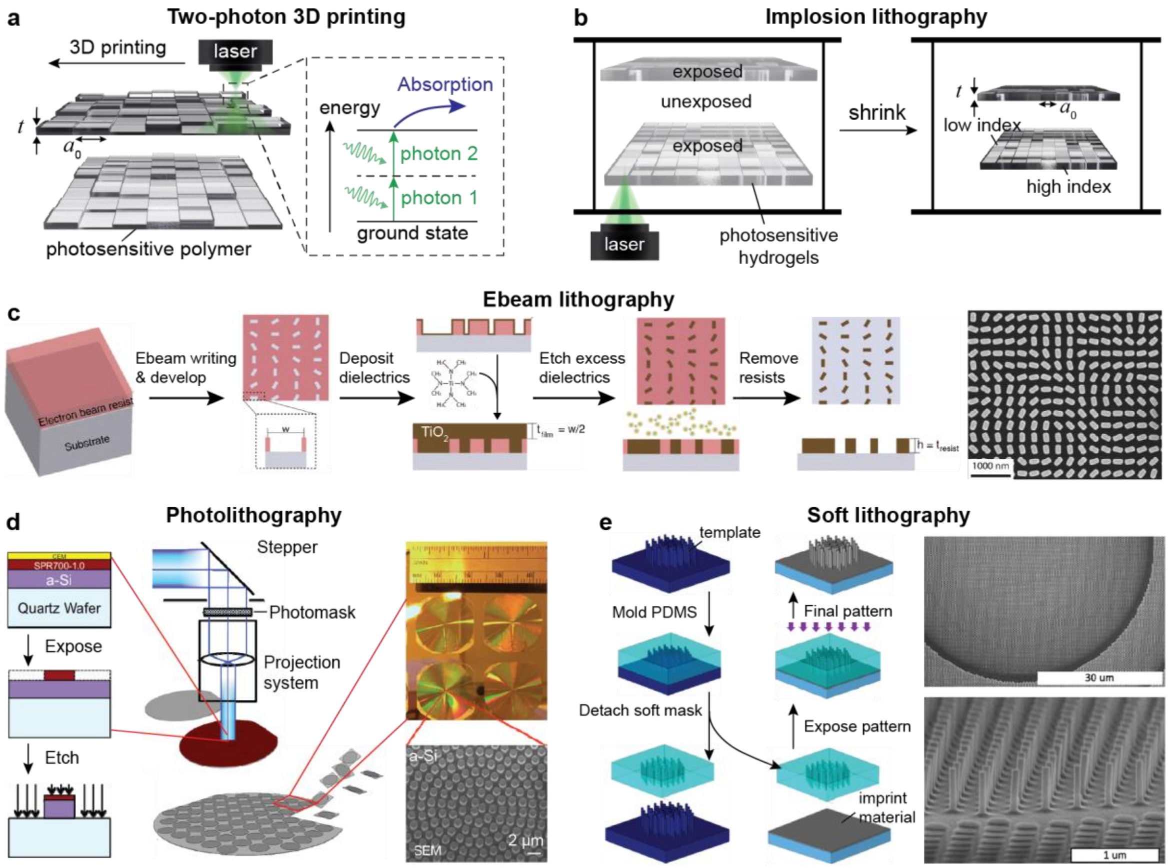

Figure S2: Fabrication methods of free-space optical processors. 3D printing techniques for fabricating diffractive networks in the visible and near-infrared wavelengths based on (**a**) two-photon laser writing[12,15] and (**b**) implosion lithography where the printed structures made of hydrogels are further shrunk by a dehydration process.[16] (**c**) Electron beam lithography for prototyping dielectric metasurfaces in the visible.[17] Scalable patterning techniques for the parallel production of dielectric metasurfaces by (**d**) photolithography[18] and (**e**) soft lithography techniques such as nanoimprinting.[19] Panel c is adapted from REF[17] with permissions from NAS. Panel d is adapted from REF[18] with permissions from The Optical Society. Panel e is adapted from REF[19] with permissions from ACS.

References:

1. Lin, X.; Rivenson, Y.; Yardimci Nezih, T.; Veli, M.; Luo, Y.; Jarrahi, M.; Ozcan, A., All-Optical Machine Learning Using Diffractive Deep Neural Networks. *Science* **2018,** *361*, 1004-1008.
2. Ocier, C. R.; Richards, C. A.; Bacon-Brown, D. A.; Krueger, N. A.; Clawson, M. K.; Soares, J. A. N. T.; Braun, P. V., Optically Anisotropic Porous Silicon Microlenses with Tunable Refractive Indexes and Birefringence Profiles. *Opt. Mater. Express* **2020,** *10*, 868-883.
3. Kulce, O.; Mengu, D.; Rivenson, Y.; Ozcan, A., All-Optical Synthesis of an Arbitrary Linear Transformation Using Diffractive Surfaces. *Light: Science & Applications* **2021,** *10*, 1-21.
4. Huang, L.; Chen, X.; Mühlenbernd, H.; Li, G.; Bai, B.; Tan, Q.; Jin, G.; Zentgraf, T.; Zhang, S., Dispersionless Phase Discontinuities for Controlling Light Propagation. *Nano Letters* **2012,** *12*, 5750-5755.
5. Khorasaninejad, M.; Chen, W. T.; Devlin, R. C.; Oh, J.; Zhu, A. Y.; Capasso, F., Metalenses at Visible Wavelengths: Diffraction-Limited Focusing and Subwavelength Resolution Imaging. *Science* **2016,** *352*, 1190-1194.
6. Khorasaninejad, M.; Zhu, A. Y.; Roques-Carmes, C.; Chen, W. T.; Oh, J.; Mishra, I.; Devlin, R. C.; Capasso, F., Polarization-Insensitive Metalenses at Visible Wavelengths. *Nano Letters* **2016,** *16*, 7229-7234.
7. Kwon, H.; Sounas, D.; Cordaro, A.; Polman, A.; Alù, A., Nonlocal Metasurfaces for Optical Signal Processing. *Physical review letters* **2018,** *121*, 173004.
8. Song, J.-H.; van de Groep, J.; Kim, S. J.; Brongersma, M. L., Non-Local Metasurfaces for Spectrally Decoupled Wavefront Manipulation and Eye Tracking. *Nature Nanotechnology* **2021,** *16*, 1224-1230.
9. Overvig, A.; Alù, A., Diffractive Nonlocal Metasurfaces. *Laser & Photonics Reviews* **2022,** *16*, 2100633.
10. Ra'di, Y.; Sounas, D. L.; Alù, A., Metagratings: Beyond the Limits of Graded Metasurfaces for Wave Front Control. *Physical Review Letters* **2017,** *119*, 067404.
11. Cordaro, A.; Edwards, B.; Nikkhah, V.; Alù, A.; Engheta, N.; Polman, A., Solving Integral Equations in Free Space with Inverse-Designed Ultrathin Optical Metagratings. *Nature Nanotechnology* **2023**.
12. Anscombe, N., Direct Laser Writing. *Nature Photonics* **2010,** *4*, 22-23.
13. Balli, F.; Sultan, M.; Lami, S. K.; Hastings, J. T., A Hybrid Achromatic Metalens. *Nature Communications* **2020,** *11*, 3892.
14. Qin, F.; Ding, L.; Zhang, L.; Monticone, F.; Chum, C. C.; Deng, J.; Mei, S.; Li, Y.; Teng, J.; Hong, M.; Zhang, S.; Alù, A.; Qiu, C.-W., Hybrid Bilayer Plasmonic Metasurface Efficiently Manipulates Visible Light. *Science Advances* **2016,** *2*, e1501168.
15. Fullager, D. B.; Boreman, G. D.; Hofmann, T., Infrared Dielectric Response of Nanoscribe Ip-Dip and Ip-L Monomers after Polymerization from 250 Cm^{-1} to 6000 Cm^{-1}. *Opt. Mater. Express* **2017,** *7*, 888-894.
16. Oran, D.; Rodriques Samuel, G.; Gao, R.; Asano, S.; Skylar-Scott Mark, A.; Chen, F.; Tillberg Paul, W.; Marblestone Adam, H.; Boyden Edward, S., 3d Nanofabrication by Volumetric Deposition and Controlled Shrinkage of Patterned Scaffolds. *Science* **2018,** *362*, 1281-1285.
17. Devlin, R. C.; Khorasaninejad, M.; Chen, W. T.; Oh, J.; Capasso, F., Broadband High-Efficiency Dielectric Metasurfaces for the Visible Spectrum. *Proceedings of the National Academy of Sciences* **2016,** *113*, 10473-10478.

18. She, A.; Zhang, S.; Shian, S.; Clarke, D. R.; Capasso, F., Large Area Metalenses: Design, Characterization, and Mass Manufacturing. *Opt. Express* **2018,** *26*, 1573-1585.
19. Einck, V. J.; Torfeh, M.; McClung, A.; Jung, D. E.; Mansouree, M.; Arbabi, A.; Watkins, J. J., Scalable Nanoimprint Lithography Process for Manufacturing Visible Metasurfaces Composed of High Aspect Ratio Tio2 Meta-Atoms. *ACS Photonics* **2021,** *8*, 2400-2409.
